# Effectiveness, relapse prevention and mechanisms of change of cognitive therapy vs. interpersonal therapy for depression: Study protocol for a randomised controlled trial

**DOI:** 10.1186/1745-6215-12-150

**Published:** 2011-06-14

**Authors:** Lotte HJM Lemmens, Arnoud Arntz, Frenk PML Peeters, Steven D Hollon, Anne Roefs, Marcus JH Huibers

**Affiliations:** 1 Department of Clinical Psychological Science, Faculty of Psychology and Neuroscience, Maastricht University, the Netherlands; 2 Department of Psychiatry and Neuropsychology, Maastricht University Medical Centre, Maastricht, the Netherlands; 3 Vanderbilt University, Department of Psychology, Nashville, Tennessee, USA

## Abstract

**Background:**

Major depression is a common mental disorder that substantially impairs quality of life and has high societal costs. Although psychotherapies have proven to be effective antidepressant treatments, initial response rates are insufficient and the risk of relapse and recurrence is high. Improvement of treatments is badly needed. Studying the mechanisms of change in treatment might be a good investment for improving everyday mental health care. However, the mechanisms underlying therapeutic change remain largely unknown. The objective of the current study is to assess both the effectiveness of two commonly used psychotherapies for depression in terms of reduction of symptoms and prevention of relapse on short and long term, as well as identifying underlying mechanisms of change.

**Methods:**

In a randomised trial we will compare (a) Cognitive Therapy (CT) with (b) Interpersonal therapy (IPT), and (c) an 8-week waiting list condition followed by treatment of choice. One hundred eighty depressed patients (aged 18-65) will be recruited in a mental health care centre in Maastricht (the Netherlands). Eligible patients will be randomly allocated to one of the three intervention groups. The primary outcome measure of the clinical evaluation is depression severity measured by the Beck Depression Intenvory-II (BDI-II). Other outcomes include process variables such as dysfunctional beliefs, negative attributions, and interpersonal problems. All self-report outcome assessments will take place on the internet at baseline, three, seven, eight, nine, ten, eleven, twelve and twenty-four months. At 24 months a retrospective telephone interview will be administered. Furthermore, a rudimentary analysis of the cost-effectiveness will be embedded. The study has been ethically approved and registered.

**Discussion:**

By comparing CT and IPT head-to-head and by investigating multiple potential mediators and outcomes at multiple time points during and after therapy, we hope to provide new insights in the effectiveness and mechanisms of change of CT and IPT for depression, and contribute to the improvement of mental health care for adults suffering from depression.

**Trial registration:**

The study has been registered at the Netherlands Trial Register, part of the Dutch Cochrane Centre (ISRCTN67561918)

## Background

With a lifetime prevalence of 17%, depression is a major health problem with serious clinical and social consequences. It is expected that depression will be the leading global cause of years of health lost due to disease in 2030 [1]. With initial response rates up to 60%, and the majority of patients regaining their normal level of functioning within three years, certain psychotherapies and antidepressant medication have proven efficacy in treating acute Major Depressive Disorder (MDD) [2,3]. This might sound promising, but at least 40% of depressed patients do not respond to initial treatment at all. Furthermore, depression has an unfavourable prognosis; even when treated effectively in the acute phase, recovery is often incomplete, which increases the chance of relapse and recurrence up to 87% over 15 years [4-7].

With this in mind, the challenge in contemporary depression research is to improve treatments to increase acute response rates and prevent relapse and recurrence in the long term. Many researchers agree that knowledge of underlying mechanisms that can explain therapeutic change is a key to improving treatment [8,9]. Knowing how a therapy works would allow honing its components to make it more efficient and (cost-)effective [10]. The current study will focus on the effectiveness, prevention of relapse and recurrence and mechanisms of change of two commonly used types of psychotherapy for depression: Cognitive Therapy (CT) [11] and Interpersonal Therapy (IPT) [12]. A rudimentary analysis of the cost-effectiveness from a societal perspective will be embedded.

### Effectiveness

Of the psychotherapeutic interventions for depression, CT and IPT might be the two best studied and empirically validated [13-15]. They come from different theoretical backgrounds: CT derives from Beck's cognitive theory and explains depression as a result of maladaptive information-processing, whereas IPT links depressive episodes to distressing life events and insufficient social support [12,16]. Nonetheless, both therapies have proven to be well-standardized, efficacious treatments for acute treatment of MDD [3,12,17-23]. There is no consensus yet about whether the effect of one therapy outperforms the other. Many studies have investigated the effects of CT and IPT separately, but only 2 large studies have compared them head-to-head [24-26]. However, doubts have arisen about the validity of one of these studies because analysis of treatment adherence showed no contrast between the two intervention groups [24,27]. Thus, the current view is based on only one methodologically well-designed study. Therefore, there is a need for renewed head-to-head comparisons of both therapies.

### Relapse Prevention

In addition to the fact that CT and IPT have shown to be efficacious acute treatments of MDD, they may also reduce the risk of relapse (episode of MDD after remission) and recurrence (episode of MDD after recovery) in the long term. The effects and evidence differ for the two therapies. Research has shown that CT has an enduring effect that extends beyond the end of therapy [12,28-33], thereby reducing the chances of relapse and recurrence. The evidence for this is strong and consistent [31,34,35]. However, the long term effect of IPT has not been tested extensively yet. Up until now it has only been tested as a maintenance treatment [36,37], and the question remains whether IPT also has an enduring effect that remains after therapy is finished. This question should be further explored. Insight in the long term effects of IPT furthermore creates the opportunity to compare CT and IPT to assess whether one therapy is superior to the other in preventing relapse and recurrence in the long term [15].

### Mechanisms of Change

As noted above, insight into mechanisms of change might contribute to the process of therapy improvement. However, the mechanisms that cause therapeutic change are still largely unknown. Despite several research attempts to identify the mechanisms of change in psychotherapy, no study has identified a model that explains change in CT or IPT completely [38,39]. Mechanism research has to cope with several methodological and theoretical difficulties [40,41]. Theoretical difficulties arise because there are conflicting hypotheses on the mechanisms and there is no consensus about the most important causes of change [38,42]. For example, it is unclear whether therapeutic change can be better explained by change in treatment specific factors [42-45] or non-specific (common) factors [46-48]. Specific factors are elements marked as the active causes of change in the theory of the therapy, such as change in cognitions in CT and optimization of interpersonal functioning in IPT. Non-specific factors refer to elements in a therapy that contribute to improvement, but that are common to all psychotherapies, such as expectancy and therapeutic alliance [49]. Furthermore, it is not exactly known whether changes achieved in therapy are best reflected by explicit or by implicit measures of psychopathology. Explicit measures depend on introspection, and a disadvantage is that it is known that people do not have access to all of the mechanisms that underlie their behaviour [50]. An implicit measure is defined by De Houwer, Teige-Mocigemba, Spruyt, and Moors (2009) as "a measurement outcome that is causally produced by the to-be-measured attribute in the absence of certain goals, awareness, substantial cognitive resources, or substantial time" (p. 350) [51]. To the extent that implicit measures reflect uncontrollable, unaware, fast mechanisms, they could provide information that augments that from explicit measures [52].

Furthermore, methodological difficulties arise because many study designs do not meet the criteria for reputable mechanisms research [40,41]. Theories often explain change in terms of causal processes. However, in many studies it is difficult to identify temporal relationships in order to investigate these causal pathways because of the absence of an appropriate time line and assessment on multiple time points [40,41]. It is clear that there is a need for renewed, methodologically well-considered mechanism research.

The question remains what is necessary for proper mechanism research. According to Kazdin (2007), a first step into investigating mechanisms of change is studying mediating variables [40]. A mediator explains why and in what way a treatment has an effect on the outcome, and plays a crucial role in the development of causal pathways. In identifying mediators, Kazdin has built upon the MacArthur guidelines of Kraemer et al. (2001) [53] which are based on the more traditional guidelines for statistical mediation formulated by Baron and Kenny (1986) [54]. In addition to statistical mediation and association, Kazdin emphasizes the importance of the temporal relationship, and consistency and specificity of the mediator. The importance of the aspect of temporality is also emphasized by Murphy et al (2009) [55]. Taking this into account, the current study will investigate potential mediators of CT for depression and test their specificity in comparison to IPT, and vice versa, by measuring multiple potential mediators and outcomes at multiple time points during and after therapy. This method enables us to investigate temporal relationships between changes in potential mediators and symptom reduction and to assess whether change in a mediator precedes, follows from, or goes together with changes in depression. In addition, this method can show us whether change in one mediator precedes change in another mediator.

### Main research questions and Hypotheses

The following main research questions were formulated:

• Are CT and IPT effective interventions in treating the acute phase of MDD and is one therapy superior to the other?

• What are the underlying psychological mechanisms of change in CT and IPT and are these mechanisms therapy-specific?

• Are CT and IPT effective in preventing relapse or recurrence of MDD in the long-term? Is one therapy superior to the other, and if so, how can these preventive effects be explained?

In line with previous research, it is hypothesized that the amount of change in depressive symptoms after therapy will be similar in both the CT and the IPT group, indicating that both treatments are just as effective in treating depression in the acute phase [14,24,56,57]. Because both therapies originally stem from different theoretical backgrounds, we expect that both treatments target depression through different key processes. It is expected that changes in cognitive schemas, attitudes, and cognitions are the most significant contributors to symptom change in CT, whereas in IPT it is assumed that improvement of interpersonal functioning will lead to a reduction of symptoms [12,16]. With regard to the mechanisms of change, many hypotheses are possible, especially when it comes to the order of change and causal pathways that lead to recovery. Following the theories one would expect that change in cognitions (CT) and interpersonal functioning (IPT) precede symptom change. Furthermore, we expect that it is the direct comparison of explicit versus implicit measures that will elicit new insights into the underlying mechanisms of change. To the extent that implicit measures reflect uncontrollable, unaware and fast mechanisms, they can provide useful additional information as compared to explicit measures. As to the prevention of relapse, we hypothesize that both CT and IPT may reduce the risk of relapse and recurrence in the long term. However, we expect CT to prevent relapse to a greater extent, because it has shown to have an enduring effect that extends beyond the end of therapy, while IPT so far only seems to be effective in treating depression as long as the treatment is continued [58].

## Methods

### Design of the study

**Figure 1:**
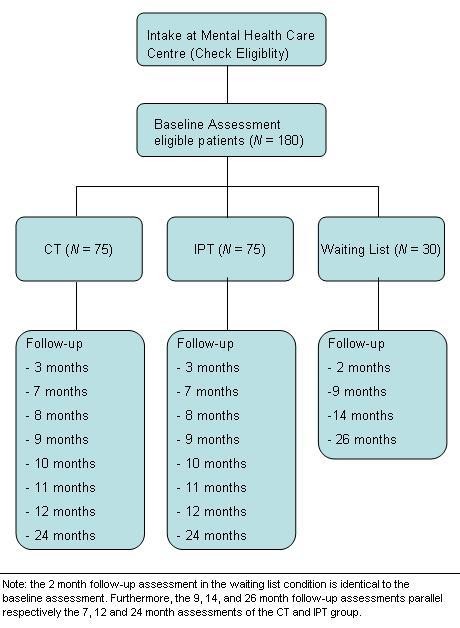
**Flow of the Participants**.

We will conduct a randomised controlled trial (RCT) in which participants will be allocated to one of three conditions: (a) CT (*N *= 75), (b) IPT (*N *= 75), (c) or a waiting list condition followed by treatment of choice (*N *= 30). Participants allocated to the waiting list condition start their treatment after an 8-week waiting period. To compensate for the waiting, they may choose their preferred treatment (CT or IPT). The anticipated flow of subject enrolment is graphically shown in Figure 1. The Medical Ethics Committee of Maastricht University approved the study protocol. The study is registered at the Netherlands Trial Register, part of the Dutch Cochrane Centre (ISRCTN67561918).

### Participants

The population we aim to investigate consists of 180 depressed adults. Patients will be eligible to participate if they meet the following criteria: age between 18 - 65 years old; the presence of a depressive episode as indicated by a diagnosis on the Structural Clinical Interview for DSM-IV Axis I disorders (SCID-I) [59]; internet access and an e-mail address; sufficient knowledge of the Dutch language; no chronic depression (>5 years); no current use of antidepressant mediation; no severe psychiatric co-morbidity, drugs- and alcohol abuse/dependence and oligophrenia.

### Sample size

To calculate the sample size of the CT and IPT group, we combined rudimentary findings from previous randomised trials, which resulted in a mean relapse/recurrence rate of 33% after two years of follow-up, compared to 67% following antidepressant medication and/or clinical management [28,29,31-33]. Based on these results, we assume a difference of 20% in relapse between CT and IPT. Using survival analysis (two-sided, α = 0.05, power = 80%), we calculated that 75 patients per treatment condition would suffice to detect this statistically significant difference rate in relapse/recurrence between the two conditions in the long term.

A waiting list condition is included to investigate whether the effects of CT and IPT exceed the outcome in patients who do not receive treatment, a finding that has been demonstrated in numerous previous studies [60-63]. A power calculation showed that 30 participants in the waiting list condition will be sufficient to detect a statistically significant difference between the two active conditions combined (CT and IPT) and the waiting list after eight weeks.

### Recruitment

Participants will be recruited during regular intakes at our clinical site, the Academic Community Mental Health Centre (RIAGG) Maastricht. The SCID-I will be administered to assess current and lifetime episodes of Axis I disorders [59]. Patients meeting the inclusion criteria will be approached for participation. If they agree to participate, the SCID for Axis II disorders (SCID-II) will be administered as well [64]. After inclusion, the participants' GP will be informed about his or her participation in the study. Participants who do not meet the inclusion criteria will be offered suitable alternative treatment options.

### Randomisation and procedure

Eligible participants will be contacted by telephone for a baseline visit at the university. Preceding this initial visit to the research centre, participants will receive an appointment letter, including study information. This will allow them to consider their study participation well before they agree to participate (approximately one week). Furthermore, to decrease the burden of the first assessment, participants will be asked to already fill out a substantial part of the baseline questionnaires at home. Randomisation will take place after informed consent is obtained and the second part of the baseline questionnaires are filled out. In our design we prestratified groups by first (1 episode) and recurrent (> 1 episodes) depression. The researcher will press the 'assign' button on the computer screen, after which the database randomly allocates the participant to one of the treatment conditions (CT or IPT), or to the waiting list condition by using block randomisation. This waiting list condition is included because it has been frequently argued in psychotherapy research that the effect in treatment studies cannot be attributed to the intervention(s) that are studied, unless a placebo or no-treatment group is included [65]. With regard to the nature of the treatments, blinding of the participants and researchers is not possible.

In reporting the follow-up period of the CT and IPT condition in this study, we distinguish three phases; (a) the Treatment phase (baseline to 7 months); the period when therapy will be delivered; (b) The Trial follow-up phase (month 8-12) in which depression severity will be measured monthly; and (c) The Long-term follow-up phase (12 to 24 months); the period covered by retrospective assessment. All questionnaires will be administered on a computer. Pre- and post treatment assessment (baseline and 7 months) will take place at the University. All other follow-up self-report assessments will take place at home via the internet at three, eight, nine, ten, eleven, twelve and twenty-four months after inclusion.

Although the baseline assessment is the same, the follow-up period of the waiting list condition is somewhat different as compared to the CT and IPT condition (see Figure 1). Participants in this condition will be measured less often, and at different points in time. The treatment phase of the participants in this condition does not start immediately after baseline, but after an 8-week waiting list period. To be able to investigate the effect of this waiting period, and to mark the starting point of the treatment phase, a second identical baseline assessment will take place two months after the initial baseline assessment. As a consequence, the post-treatment assessment in this condition will take place after nine months (instead of after seven months in the CT and IPT condition). Furthermore, patients in this condition will only have two more assessment after their post-treatment measure. This will take place at 14 and 26 months, and is identical to the 12 and 24 months assessment of the CT and IPT condition. To summarize, patients in the waiting list condition will have the same baseline assessment as compared to participants in the CT and IPT condition. However, they will have a second baseline assessment at two months follow-up, and their 9, 14, and 26 month follow-up assessments will parallel respectively the 7, 12, and 24 months assessments of the CT and IPT group (see table 1).

**Table 1:** Overview of instruments per time point in the CT and IPT group*

Instrument	0**	3	7	8-11	12	24
						
*Clinical Outcome measures*						
Beck Depression Inventory II	**•**	**•**	**•**	**•**	**•**	**•**
Quick Inventory of Depressive symptoms	**•**				**•**	
Brief Symptom Inventory	**•**		**•**		**•**	**•**
Diagnostic Interview for Depression		**•**	**•**	**•**	**•**	**•**
Beck Hopelessness Scale	**•**		**•**			
Longitudinal Interval Follow-up Evaluation						**•**
						
*Process variables*						
Attributional Style questionnaire	**•**	**•**	**•**		**•**	
Dysfunctional Attitude Scale	**•**	**•**	**•**		**•**	
Inventory of Interpersonal Problems	**•**	**•**	**•**		**•**	**•**
Leiden Index of Depression Severity	**•**	**•**	**•**			
Ruminative Response Scale NL	**•**	**•**	**•**			
Self-Liking and Self-Competence Scale	**•**	**•**	**•**			
Single Category Implicit Association Task	**•**		**•**			
						
*Economic Evaluation measures*						
Health care use Questionnaire	**•**	**•**	**•**		**•**	**•**
Productivity and Disease Questionnaire	**•**		**•**		**•**	**•**
Work and Social Adjustment Scale	**•**		**•**		**•**	**•**
Euroqol-6D	**•**		**•**		**•**	**•**
Rand 36	**•**		**•**		**•**	**•**
						
*Other measures*	**•**					
Genetic information						
Working Alliance Inventory		**•**				
Collaborative Study Psychotherapy Rating Scale	**•**	**•**	•***			
Working Alliance Inventory-short observer version	**•**	**•**	•***			

* Patients in the waiting list condition will receive the same baseline assessment, they will have a second baseline assessment at 2 months follow-up, and their 9, 14, and 26 month follow-up assessments parallel respectively the 7, 12, and 24 month assessments of the CT and IPT group. ** The following general predictors will be administered at baseline as well: demographic variables, family anamnesis, childhood experiences, life-events, reliability and expectations. *** During the treatment phase, all sessions will be videotaped. Only a random selection of tapes will be rated.

In all conditions a BDI-II will be filled out before each session during the treatment phase. After the two-year follow-up period, a modified version of the semi-structured Longitudinal Interval Follow-Up Evaluation (LIFE) [66] will be administered to retrospectively map out the course of depressive symptoms. To optimize the adherence of self-report assessments, participants will receive an email foregoing each assessment point. If one does not complete the questionnaires within one week, an email-reminder will be sent. If the participant still fails to fill out the questionnaires, a phone call will be made.

### Interventions

The interventions will be offered at the Academic RIAGG Maastricht. At this site, CT and IPT are already delivered as treatment as usual. Interventions are written out in a treatment manual, and both treatments will contain 12 to 20 sessions of 45 minutes, depending on the individual progress of patients. Interventions will be delivered by qualified therapists, who were trained by Steven Hollon (CT) and John Markowitz (IPT), experts in the field of depression treatment. To prevent contamination of treatment conditions, participating therapists will deliver either CT or IPT. Therapists will consult each other on current cases in biweekly consultation meetings. The CT protocol is based on the manual by Beck (1979) and is modified for the current study with a special focus on relapse prevention. IPT is based on the manual of Klerman, Weissman, Rounsaville, and Chevron (1984). CT and IPT differ in terms of target (cognitions and behaviour vs. interpersonal functioning), approach (directive vs. empathic-reflective) and method (homework assignment vs. no assignments) [27]. All sessions will be videotaped, and a random selection of tapes will be analysed by independent assessors on treatment adherence (content and quality) by using the Collaborative Study Psychotherapy Rating Scale - version 6 (CSPRS-6) [67], and on quality of therapeutic alliance using the Observer version of the Working Alliance Inventory-Short (WAI-O-S) [68-72].

### Instruments

Several instruments will be used to assess the effects of CT and IPT on depressive symptoms and other secondary outcomes. Furthermore, instruments will be used to assess the mechanisms of change and the cost-effectiveness of CT and IPT. An overview of all measurements per assessment is given in table 1.

### Clinical outcome measures

#### Beck Depression Inventory-II

The Beck Depression Inventory-II (BDI-II) [73,74] will be used as the primary outcome measure of depressive severity. The total score is the sum of the 21 items ranging from 0 to 63. Higher scores reflect more severe depressive symptoms. Several studies have shown that the BDI-II is a strong screening measure for depression [73-75].

#### Quick Inventory of Depressive Symptoms

The Quick Inventory of Depressive Symptoms (QIDS) [76] is a treatment sensitive measure of symptom severity in depression. The 16 items that measure 9 criterion domains of MDD are derived from the 30-item Inventory of Depressive Symptomatology (IDS) [77]. The QIDS has been shown to have highly acceptable psychometric properties [78].

#### Brief Symptom Inventory

The Brief Symptom Inventory (BSI) [79] is a 53-item self-report instrument that will be used to measure general psychological distress. It is derived from the SCL-90-R and several studies have demonstrated it to be an acceptable short alternative of its longer version [80,81].

#### Diagnostic Inventory for Depression

The Diagnostic Inventory for Depression (DID) will be used to assess the psychosocial impairment due to depression, and to evaluate subjective quality of life [82,83]. Psychometric analysis shows good reliability and validity [82].

#### Longitudinal Interval Follow-up Evaluation

The modified version of the semi-structured Longitudinal Interval Follow-up Evaluation (LIFE) [66] will be used to map out the course of depressive symptoms in the long-term follow-up phase (12-24 months). This will be administered at the end of the follow-up (24 months). We used the MDD section of the original interview, and added items on general functioning, working status, relations to others and treatment- and study experiences. The LIFE has shown to be a reliable and valid instrument for characterizing the course of several mental disorders examined retrospectively over the period of one year [84,85].

### Process (or mechanism) variables

#### Dysfunctional Attitudes

The Dysfunctional Attitude scale version a (DAS-A) [86-88] is a self-report scale designed to measure patterns of negative and absolute thinking in depressed patients. Respondents need to report whether or not they agree with a series of dysfunctional assumptions on a seven point Likert-Scale. With its good internal consistency and validity, it appears to be a valid measure of dysfunctional cognitions in depressed patients [89-92].

#### Attributional Style

Attributional style will be measured using the Attributional Style Questionnaire (ASQ) [93,94]. In the ASQ, participants receive 12 hypothetical events and are asked to imagine themselves in that scenario. Subsequently they have to decide what the major cause of the situation would be if it happened to themselves and rate the cause along a 7-point Likert-scale on three dimensions: Personal, Permanent and Pervasive. Depressive symptoms are associated with an attributional style in which the causes of good and bad events are attributed to internal (versus external), stable (versus unstable), and global (versus specific) causes [95]. It is hypothesized that if attributional style changes significantly during therapy, the explanations of why events happen change to external, unstable and specific. The ASQ has shown to have good psychometric properties [93].

#### Implicit measure of self-esteem

A variant of the Single Category Implicit Association Test (Sc-IAT) [96] will be administered to obtain an implicit measure of associations with the self (self-esteem). For the current study, we adapted the original Sc-IAT paradigm to specifically measure feelings of helplessness, unlovability and worthlessness, which are considered the main themes of core beliefs in depression. The test consists of 3 blocks of trials and starts with a practice block. In this block, positive and negative words are presented (all words are related to the specific core beliefs), and the task for participants is to categorize these stimuli as such by pressing the appropriate key (left or right) without making too many errors. After the practice block, there are two test blocks. In one critical block, stimuli representing the self ('me-trials') share a response key with positive stimuli, while the other key needs to be pressed for negative stimuli. In the other critical block, the target key assignment for me-trials is switched. Stimuli representing the self now share a response key with the negative stimuli. Each block is preceded by a set of instructions concerning the dimension(s) of the categorisation task and the appropriate key response. The rationale behind the Sc-IAT is that the response time will be faster when two associated concepts are mapped together on the same key, compared to when two less compatible concepts are mapped together. For example, in people with a positive sense of self-esteem, when 'valuable' and 'me' meaning words are mapped to the same key, the response time will be faster than for the non-compatible combination ('worthless' and 'me'). The Sc-IAT effect will be calculated using the improved D-Algorithm [97]. In comparison with implicit measures obtained via other measurement procedures, the Sc-IAT shows good psychometric qualities [98-101].

#### Hopelessness

The Beck Hopelessness Scale (BHS) [102] is a self-report instrument that determines the extent of positive and negative beliefs about the future over the previous 7 days using 20 true-false statements. All statements are scored 0 or 1 with the total being calculated by summing the pessimistic responses for the items. General cut-off guidelines do not exist and it is recommended that cut-off scores should be based upon clinical decisions. The BHS has been shown to have good psychometric properties for application in clinical samples [103-105].

#### Interpersonal Problems

The Inventory of Interpersonal Problems (IIP) [106] is a 127-item self-report measure, which describes the types of problems that people experience in their relationships with others, and the level of distress associated with them. Items are divided in two sections: participants are asked to rate problematic behaviour that is hard for them to do (78 items) and behaviour that they are doing too much (49 items). The IIP provides a total score and scores on six or eight subscales. Higher scores indicate more interpersonal problems. Psychometric properties of the IIP were found to be good [106].

#### Cognitive Reactivity

During a depressive episode, an association is formed between depressed mood and dysfunctional thinking. This association may remain intact even when the depressive episode remits. The strength of this association is called cognitive reactivity [107]. The Leiden Index of Depression Sensitivity (LEIDS) is used to measure this cognitive reactivity on six subscales [108]. Psychometric qualities of this measurement are found to be good [108].

#### Rumination

The Ruminative Response Scale (RRS) [109,110] is a 22-item questionnaire that will be used to detect the responses to depressed mood that are focused on self, symptoms, or possible consequences and causes of mood. People are asked to indicate how often they think about certain things on a four point Likert-scale. The RRS shows good reliability and satisfactory validity [109,110].

#### Self-Esteem

The Self Liking and Self Competence Scale Revised (SLCS-R) [111,112] is a self-report questionnaire constructed to measure Self-Competence and Self-Liking, two dimensions of self-esteem. The SLSC-R contains eight items for each of the two dimensions. Items are rated on a 5-point Likert scale. The subscales scores can range from 8 to 40, with higher scores indicating higher self-competence or higher self-liking. Psychometric properties were found to be good [111].

### Economic Evaluation

#### Costs

A rudimentary analysis of the cost-effectiveness of CT and IPT will be conducted from a societal perspective. We will distinguish three cost categories: health care sector costs, costs for the patient and family, and productivity cost [113]. Healthcare costs and patient and family costs will be measured using a periodic retrospect health care consumption questionnaire designed by de Graaf et al. (2008) for use in the field of depression research [114]. This questionnaire is based on an existing cost diary [115] and retrospective cost questionnaires [116,117]. Containing 52 items, it measures the number and content of contacts with GPs, specialists, paramedics, alternative care, psychological care, hospital visits, medication, and self-help over a period of three months. To measure production losses we will use the patient modules (A-E) of the productivity and disease questionnaire (PRODISQ) [118].

#### Quality of Life measures

To measure the experiential impact of the disorder, the Work and Social Adjustment Scale (W&SAS) [119] will be used. This 5-item self-report scale examines to what extent the disorder impairs a person's everyday functioning. Items are rated on a 9 point Likert-scale, with higher scores indicating more severe impairment. Psychometric properties of the W&SAS are found to be good [119,120]. Furthermore, to measure generic quality of life of the patients on the basis of societal appreciation of health condition, the EuroQol [121] will be used. To measure quality of life in terms of impairments due to physical and mental health status, we will use the RAND-36 [122]. This 36 item questionnaire measures physical and social functioning, role restriction due to physical or emotional problems, mental health, energy, pain, and general health perception. The RAND-36 has shown to be a reliable, valid and sensitive measure [122].

### Other measures

#### Working Alliance

The past decades, it has become clear that therapeutic alliance is an essential element of the therapeutic process [123]. The stronger the collaborative and affective bond between patient and therapist, the larger the therapeutic change [124]. To measure this bond, the Working Alliance Inventory (WAI) [69,125] will be used. The questionnaire consists of 36 items rated on a 5-point Likert scale and will be filled out by both patient and therapist. The internal consistency of this instrument was found to be high. As has been mentioned earlier, all therapy sessions will be videotaped. Therapeutic alliance will also be assessed by rating a random selection of videotapes using the Observer version of the Working Alliance Inventory-Short (WAI-O-S) [71,72].

#### Genetics

There is evidence that the same genetic factors that appear to moderate the efficacy of different classes of antidepressants are also involved in aspects of cognitive functioning [126]. The same polymorphisms are also associated with reactions on aversive stimuli [127-129]. Because changes in regulation of emotions and cognitive processes are core elements in therapeutic approaches such as CT and IPT, we expect that the efficacy of these therapies is influenced by genetic variations in polymorphisms. Therefore, we will obtain genetic material at baseline (buccal cotton swabs).

### Analyses

Data-analysis will include intention-to-treat analyses, per-protocol analyses, change analyses and prognostic analyses. Analyses will include elementary head-to-head comparisons of the intervention groups as well as more complicated multivariate analyses (e.g., individual time series analysis, logistic regression analysis, multilevel analyses). Furthermore, in ancillary analysis, subgroups of the study sample according to symptom severity and DSM-IV classification will be examined for differential effects. In addition, we will determine the proportion of patients that show reliable and clinically significant improvement on the outcome measures. Our calculations will be based on the method of Jacobson and Truax [130] which prescribes that Clinical Improvement (CI) is based on both Reliable Change (RC), the extent to which the pre-to-post-difference score is reliable; and on Clinical Significant change (CSC), the extent to which post-treatment scores are clinically meaningful [131]. We will use chi-square tests to test the frequency differences in the RC, CSC, and CI between the three intervention groups.

Relapse (episode of MDD after remission) and recurrence (episode of MDD after recovery) in the course of follow-up (12 and 24 months) will be assessed using survival analysis (Cox proportional hazards regression). Furthermore, using multi-level mediation models, we will investigate various potential treatment mediators (psychological processes responsible for therapeutic change) to identify mechanisms of change. We will use the revised and expanded McArthur guidelines with a central focus on the temporal aspect [40,41]. Furthermore, we will use structural equation modelling to test the relative strength of the factors involved (path analysis), an approach already successfully applied by Oei and colleagues [48].

## Discussion

We presented the protocol of a study assessing the effectiveness, mechanisms of change and prevention of relapse/recurrence of CT and IPT for depression. While it is well known that both CT and IPT are effective treatments for depression, it is not entirely clear yet whether one therapy is superior to the other, especially in the long term. Furthermore, since response rates of both therapies are insufficient and the risk of relapse and recurrence is high, the challenge in contemporary depression research is the improvement of treatments to increase response rates and prevent relapse and recurrence. Studying the mechanisms of change in treatment might be a first step in improving every day clinical practice. Although in the past decades attention for mechanisms of change in psychotherapy has grown, this type of research is still in its infancy. It may be clear that there is a need for renewed methodologically well-considered research. With the current study, we hope to provide new insights in the issues stated above.

### Methodological Considerations

The current study has several strengths, including randomisation of patients to three conditions (CT, IPT and a waiting list condition). The used design (RCT) is not only the standard for the evaluation of effectiveness of psychiatric treatments, but is also very valuable in studying mechanisms of therapeutic change [132,133]. Furthermore, we follow the recommendation of Kazdin to measure multiple potential mediators simultaneously and to include multiple follow-up measures at various time-points throughout a 2-year follow-up period. We assess both mediators and outcomes before and after therapy. This repeated measures design will provide a unique opportunity to evaluate whether change in cognitions occurs in advance of, and is related to, reduction in symptoms. In addition, to our knowledge, we are the first to compare CT with IPT head-to-head for relapse prevention and assess the underlying mechanisms of change using both explicit and implicit measures in a treatment study of depression to examine the level of therapeutic changes. To conclude, we will use state-of-the art statistical techniques to analyze temporality, causality and mechanisms of change.

However, the current study also has a number of limitations. In spite of the fact that we consider many potential mechanisms, there probably will be important (latent) processes that are not assessed in the current study. Even though we will use analysing techniques to prevent these biases as much as possible, there still is a chance that results will be contributed to the measured variables, whereas they are actually caused by other latent variables.

### Conclusions

The current study will compare CT and IPT head-to-head in terms of effectiveness and the prevention of relapse. Furthermore, in order to investigate mechanisms of change, we will investigate potential mediators of CT for depression and test their specificity in comparison to IPT and vice versa. By including assessments on multiple time points in both the treatment- and follow-up phase, we try to investigate temporal relationships between change in potential mediators and outcome measures that are needed to identify causal pathways of therapeutic change. We will assess both specific and common treatment factors using both explicit and implicit measures. We hope to provide new insights in the mechanisms of change of CT and IPT for depression and through here contribute to the improvement of health care for adults suffering from depression.

## Abbreviations

ASQ: Attributional Style Questionnaire; BDI-II: Beck Depression Inventory, second edition; BHS: Beck Hopelessness Scale; BSI: Brief Symptom Inventory; CI: Clinical Improvement; CSC: Clinical Significant Change; CSPRS-6: Collaborative Study Psychotherapy Rating Scale - version 6; CT: Cognitive Therapy; DAS-A: Dysfunctional Attitude scale version a; DID: Diagnostic Inventory for Depression; DSM-IV: Diagnostic and Statistical Manual of Mental Disorders, fourth edition; IDS: Inventory of Depressive Symptomatology; IIP: Inventory of Interpersonal Problems; IPT: Interpersonal Therapy; LEIDS: Leiden Index of Depression Sensitivity; LIFE: Longitudinal Interval Follow-Up Evaluation; MDD: Major Depressive Disorder; PRODISQ: productivity and Disease questionnaire; QIDS: Quick Inventory of Depressive Symptoms; RC: Reliable Change; RCT: Radomised Controlled Trial; RIAGG: Regional Academic Community Mental Health Centre; RRS: Ruminative Response Scale; Sc-IAT: Single Category Implicit Association Test; SCID-I: Structural Clinical Interview for DSM-IV Axis I disorders; SCID-II: Structural Clinical Interview for DSM-IV Axis II disorders; SLSC-R: Self Liking and Self Competence Scale Revised; W&SAS: Work and Social Adjustment Scale; WAI: Working Alliance Inventory; WAI-O-S: Observer rated version of the Working Alliance Inventory Short

## Competing interests

The authors declare that they have no competing interests.

## Authors' contributions

All authors, except for AR and LHJML, participated in the design of the study. MJHH obtained funding for the study. LHJML drafted the manuscript and carries out recruitment and data-collection. All authors contributed to the writing of the manuscript and have approved the final manuscript.

## Authors' information

Lotte Lemmens (MSc) is a PhD Candidate at the department of Clinical Psychological Science at the faculty of Psychology and Neuroscience of Maastricht University. Arnoud Arntz (PhD) is a professor of Clinical and Experimental Psychopathology at Maastricht University. Frenk Peeters (MD, PhD) is a psychiatrist at the Department of Psychiatry and Neuropsychology at Maastricht University Medical Centre. Steven Hollon (PhD) is a professor of Psychology at Vanderbilt University, with a cross-appointment in Psychiatry, whose research focuses on the nature and treatment of depression. Anne Roefs (PhD) is an assistant professor at Maastricht University. She is a cognitive psychologist, specialized in implicit measures. Marcus Huibers (PhD) is a professor of Empirically Directed Psychotherapy at Maastricht University. Next to scientific activities, AA, FPMLP and MJHH all work as clinicians at the Academic RIAGG Maastricht. The research group has ample experience in conducting both experimental research and clinical studies.

## References

[B1] Mathers C Fat DM Boerma JT The global burden of disease: 2004 update 2008 Geneva: World Health Organization

[B2] Roelofs J Muris P Griez EJL, Faravelli D, Nutt D, J, Zohar J Psychological treatments of depression Mood disorders: clinical management and research issues 2005 Chichester: John Wiley & Sons

[B3] Hollon SD Ponniah K A review of empirically supported psychological therapies for mood disorders in adults Depression and Anxiety 2010 27 891 932 10.1002/da.20741 20830696 PMC2948609

[B4] Beers MH Fletscher AJ Jones TV Porter R Berkwits M Kaplan JL Merck Manual 2005 Houten: Bohn Stafleu van Loghum

[B5] Keller MB Boland RJ Implications of failing to achieve successful long-term maintenance treatment of recurrent unipolar major depression Biological Psychiatry 1998 44 348 60 10.1016/S0006-3223(98)00110-3 9755357

[B6] Ormel J Oldehinkel T Brilman E Brink Wv Outcome of Depression and Anxiety in Primary Care: A Three-Wave 31/2-Year Study of Psychopathology and Disability Arch Gen Psychiatry 1993 50 759 66 8215800 10.1001/archpsyc.1993.01820220009001

[B7] Van Londen L Molenaar RPG Goekoop JG Zwinderman AH Rooijmans HGM Three- to 5-year prospective follow-up of outcome in major depression Psychological Medicine 1998 28 731 5 10.1017/S0033291797006466 9626729

[B8] Kazdin AE Nock MK Delineating mechanisms of change in child and adolescent therapy: methodological issues and research recommendations Journal of Child Psychology and Psychiatry 2003 44 1116 29 10.1111/1469-7610.00195 14626454

[B9] Kraemer HC Wilson GT Fairburn CG Agras WS Mediators and Moderators of Treatment Effects in Randomized Clinical Trials Archives of General Psychiatry 2002 59 877 83 10.1001/archpsyc.59.10.877 12365874

[B10] Warmerdam L van Straten A Jongsma J Twisk J Cuijpers P Online cognitive behavioral therapy and problem-solving therapy for depressive symptoms: Exploring mechanisms of change Journal of Behavior Therapy and Experimental Psychiatry 2010 41 64 70 10.1016/j.jbtep.2009.10.003 19913781

[B11] Beck AT Rush AJ SB F Emery G Cognitive therapy of depression 1979 New York: Guilford Press

[B12] Klerman GL Weissman MM Rounsaville BJ Chevron ES Interpersonal psychotherapy for depression 1984 New York: Basis Books

[B13] Cuijpers P Geraedts AS van Oppen P Andersson G Markowitz JC van Straten A Interpersonal Psychotherapy for Depression: A Meta-Analysis American Journal of Psychiatry 2011 168 6 581 92 Epub 2011 Mar 1 10.1176/appi.ajp.2010.10101411 21362740 PMC3646065

[B14] Cuijpers P van Straten A Andersson G van Oppen P Psychotherapy for depression in adults: A meta-analysis of comparative outcome studies Journal of Consulting and Clinical Psychology 2008 76 909 22 19045960 10.1037/a0013075

[B15] Hollon SD Thase ME Markowitz JC Treatment and prevention of depression Psychological Science and Public Interest 2002 3 39 77 10.1111/1529-1006.00008 26151569

[B16] Beck AT Rush AJ Shaw BF Emery G Cognitive therapy of depression 1979 New York: Guilford Press

[B17] Butler AC Chapman JE Forman EM Beck AT The empirical status of cognitive-behavioral therapy: A review of meta-analyses Clinical Psychology Review 2006 26 17 31 10.1016/j.cpr.2005.07.003 16199119

[B18] DeRubeis RJ Crits-Christoph P Empirically supported individual and group psychological treatments for adult mental disorders Journal of Consulting and Clinical Psychology 1998 66 37 52 9489261 10.1037//0022-006x.66.1.37

[B19] Emmelkamp PMG Velden Kvd Cognitieve gedragstherapeutische interventies bij depressie Directieve therapie 4 1992 Houten: Bohn Stafleu van Loghum

[B20] Klerman GL Weissman MM New applications of interpersonal psychotherapy 1993 Washington, DC US: American Psychiatric Association

[B21] Strunk DR DeRubeis RJ Cognitive Therapy for Depression: A Review of Its Efficacy Journal of Cognitive Psychotherapy 2001 15 289 97

[B22] Weissman MM The efficacy of drugs and psychotherapy in the treatment of acute depressive episodes The American Journal of Psychiatry 1979 136 555 8 371421

[B23] Weissman MM Markowitz JC Interpersonal psychotherapy: Current status Archives of General Psychiatry 1994 51 599 606 8042909 10.1001/archpsyc.1994.03950080011002

[B24] Elkin I Shea MT Watkins JT Imber SD National Institute of Mental Health Treatment of Depression Collaborative Research Program: General effectiveness of treatments Archives of General Psychiatry 1989 46 971 82 2684085 10.1001/archpsyc.1989.01810110013002

[B25] Luty SE Carter JD McKenzie JM Rae AM Frampton CMA Mulder RT Randomised controlled trial of interpersonal psychotherapy and cognitive-behavioural therapy for depression British Journal of Psychiatry 2007 190 496 502 10.1192/bjp.bp.106.024729 17541109

[B26] McBride C Atkinson L Quilty LC Bagby RM Attachment as moderator of treatment outcome in major depression: A randomized control trial of interpersonal psychotherapy versus cognitive behavior therapy Journal of Consulting and Clinical Psychology 2006 74 1041 54 17154734 10.1037/0022-006X.74.6.1041

[B27] Ablon JS Jones EE Psychotherapy process in the National Institute of Mental Health Treatment of Depression Collaborative Research Program Journal of Consulting and Clinical Psychology 1999 67 64 75 10028210 10.1037//0022-006x.67.1.64

[B28] Bockting CLH Schene AH Spinhoven P Koeter MWJ Wouters LF Huyser J Preventing Relapse/Recurrence in Recurrent Depression With Cognitive Therapy: A Randomized Controlled Trial Journal of Consulting and Clinical Psychology 2005 73 647 57 16173852 10.1037/0022-006X.73.4.647

[B29] Fava GA Ruini C Rafanelli C Finos L Conti S Grandi S Six-Year Outcome of Cognitive Behavior Therapy for Prevention of Recurrent Depression The American Journal of Psychiatry 2004 161 1872 6 10.1176/appi.ajp.161.10.1872 15465985

[B30] Frank E Kupfer DJ Perel JM Cornes C Three-year outcomes for maintenance therapies in recurrent depression Archives of General Psychiatry 1990 47 1093 9 2244793 10.1001/archpsyc.1990.01810240013002

[B31] Hollon SD Does cognitive therapy have an enduring effect? Cognitive Therapy and Research 2003 27 71 5 10.1023/A:1022538713914

[B32] Hollon SD DeRubeis RJ Shelton RC Amsterdam JD Salomon RM O'Reardon JP Prevention of Relapse Following Cognitive Therapy vs Medications in Moderate to Severe Depression Archives of General Psychiatry 2005 62 417 22 10.1001/archpsyc.62.4.417 15809409

[B33] Paykel ES Scott J Cornwall PL Abbott R Crane C Pope M Duration of relapse prevention after cognitive therapy in residual depression: Follow-up of controlled trial Psychological Medicine: A Journal of Research in Psychiatry and the Allied Sciences 2005 35 59 68 10.1017/s003329170400282x 15842029

[B34] Jarret RB Kraft D Doyle J Foster BM Eaves CG Silver PC Preventing recurrent depression using cognitive therapy with and without a continuation phase; a randomised clinical trial Archives of General Psychiatry 2001 58 381 8 10.1001/archpsyc.58.4.381 11296099 PMC1307495

[B35] Teasdale JD Segal ZV Williams JM Ridgeway VA Soulsby JM Lau MA Prevention of relapse/recurrence in major depression by mindfulness-based cognitive therapy Journal of Consulting and Clinical Psychology 2000 68 615 23 10965637 10.1037//0022-006x.68.4.615

[B36] Frank E Kupfer DJ Wagner EF McEachran AB Efficacy of interpersonal psychotherapy as a maintenance treatment of recurrent depression: Contributing factors Archives of General Psychiatry 1991 48 1053 9 1845438 10.1001/archpsyc.1991.01810360017002

[B37] Frank E Kupfer DJ Buysse DJ Swartz HA Pilkonis PA Houck PR Randomized trial of weekly, twice-monthly, and monthly interpersonal psychotherapy as maintenance treatment for women with recurrent depression The American Journal of Psychiatry 2007 164 761 7 10.1176/appi.ajp.164.5.761 17475735 PMC3579577

[B38] Garrat G Ingram RE Cognitive Processes in Cognitive Therapy: Evaluation of the Mechanisms of Change in the Treatment of Depression Clinical Psychology 2007 14 224 39

[B39] Longmore RJ Worrell M Do we need to challenge thoughts in cognitive behavior therapy? Clinical Psychology Review 2007 27 173 87 10.1016/j.cpr.2006.08.001 17157970

[B40] Kazdin AE Mediators and Mechanisms of Change in Psychotherapy Research Annual Review of Clinical Psychology 2007 3 1 27 10.1146/annurev.clinpsy.3.022806.091432 17716046

[B41] Kazdin AE Understanding how and why psychotherapy leads to change Psychotherapy Research 2009 19 418 28 10.1080/10503300802448899 19034715

[B42] DeRubeis RJ Evans MD Hollon SD Garvey MJ Grove WM Tuason VB How Does Cognitive Therapy Work? Cognitive Change and Symptom Change in Cogntive Therapy and Pharmacotherapy for Depression Journal of Consulting and Clinical Psychology 1990 58 862 9 2292637 10.1037//0022-006x.58.6.862

[B43] Furlong M Oei TPS Changes to automatic thoughts and dysfuntional attitudes in group CBT for depression Behavioural and Cognitive Psychotherapy 2002 30 351 60

[B44] Kwon S Oei TPS Cognitive change processes in a group cognitive behavior therapy of depression Journal of Behavior Therapy and Experimental Psychiatry 2003 34 73 85 10.1016/S0005-7916(03)00021-1 12763394

[B45] Quilty LC McBride C Bagby RM Evidence for the cognitive mediational model of cognitive behavioural therapy for depression Psychological Medicine 2008 38 1531 41 10.1017/S0033291708003772 18578895

[B46] Coleman D Cole D Wuest L Cognitive and Psychodynamic Mechanisms of Change in Treated and Untreated Depression Journal of Clinical Psychology 2010 66 215 28 19902488 10.1002/jclp.20645

[B47] Kolko DJ Brent DA Baugher M Bridge J Birmaher B Cognitive and family therapies for adolescent depression: Treatment specificity, mediation, and moderation Journal of Consulting and Clinical Psychology 2000 68 603 14 10965636

[B48] Oei TPS Bullbeck K Campbell JM Cognitive change process during group cognitive behaviour therapy for depression Journal of Affective Disorders 2006 92 231 41 10.1016/j.jad.2006.02.004 16542734

[B49] Oei TPS Shuttlewood GJ Specific and Nonspecific factors in psychotherapy; a case of cognitive therapy for depression Clinical Psychology Review 1996 16 83 103 10.1016/0272-7358(96)00009-8

[B50] Nisbett RE Wilson TD Telling more than we can know: Verbal reports on mental processes Psychological Review 1977 84 231 59

[B51] De Houwer J Teige-Mocigemba S Spruyt A Moors A Implicit measures: A normative analysis and review Psychological Bulletin 2009 135 347 68 19379018 10.1037/a0014211

[B52] Roefs A Huijding J Smulders FTY MacLeod CM de Jong PJ Wiers RW Implicit measures of association in psychopathology research Psychological Bulletin 2011 137 149 93 21219060 10.1037/a0021729

[B53] Kraemer HC Stice E Kazdin AE Offord D Kupfer DJ How Do Risk Factors Work Together? Mediators, Moderators, and Independent, Overlapping and Proxy Risk Factors American Journal of Psychiatry 2001 158 848 83 11384888 10.1176/appi.ajp.158.6.848

[B54] Baron RM Kenny DA The moderatorâ€"mediator variable distinction in social psychological research: Conceptual, strategic, and statistical considerations Journal of Personality and Social Psychology 1986 51 1173 82 3806354 10.1037//0022-3514.51.6.1173

[B55] Murphy R Cooper Z HS D Fairburn CG How do psychological treatments work? Investigating mediators of change Behavioural Research Therapy 2009 47 1 5 10.1016/j.brat.2008.10.001 PMC2726923 19010459

[B56] Dorrepaal E van Nieuwenhuizen C Schene A de Haan R De effectiviteit van cognitieve en interpersoonlijke therapie bij depressiebehandeling: een meta-analyse Tijdschrift voor Psychiatrie 1998 40 27 39

[B57] Willemse Y Trijsburg RW Cognitieve gedragstherapie en interpersoonlijke psychotherapie. Een analyse van kritische succesfactoren Tijdschrift voor Psychiatrie 2005 47 593 602

[B58] Hollon SD Jarrett RB Nierenberg AA Thase ME Trivedi M Rush AJ Psychotherapy and Medication in the Treatment of Adult and Geriatric Depression: Which Monotherapy or Combined Treatment? Journal of Clinical Psychiatry 2005 66 455 68 10.4088/JCP.v66n0408 15816788

[B59] First MB Spitzer RL Gibbon M Williams JBW Structured Clinical Interview for DSM-IV Axis I Disorders (SCID-I) 1995 New York: Biometrics Research Department, New York State Psychiatric Institute

[B60] Dobson KS A meta-analysis of the efficacy of cognitive therapy for depression Journal of Consulting and Clinical Psychology 1989 57 414 9 2738214 10.1037//0022-006x.57.3.414

[B61] Gloaguen Vr Cottraux J Cucherat M Blackburn I-M A meta-analysis of the effects of cognitive therapy in depressed patients Journal of Affective Disorders 1998 49 59 72 10.1016/S0165-0327(97)00199-7 9574861

[B62] Parker G Parker I Brotchie H Stuart S Interpersonal psychotherapy for depression? The need to define its ecological niche Journal of Affective Disorders 2006 95 1 11 10.1016/j.jad.2006.03.019 16712944

[B63] Robinson LA Berman JS Neimeyer RA Psychotherapy for the treatment of depression: A comprehensive review of controlled outcome research Psychological Bulletin 1990 108 30 49 2200072 10.1037/0033-2909.108.1.30

[B64] First MB Gibbon M Spitzer RL Williams JBW Benjamin LS Structured Clinical Interview for DSM-IV Axis II Personality Disorders (SCID-II) 1997 Washington, D.C: American Psychiatric Press

[B65] Klein DF NIMH collaborative research on treatment of depression Archives of General Psychiatry 1990 47 682 4 2163248 10.1001/archpsyc.1990.01810190082012

[B66] Keller MB Lavori PW Friedman B Nielsen E Endicott J McDonald-Scott P The Longitudinal Interval Follow-up Evaluation: A Comprehensive Method for Assessing Outcome in Prospective Longitudinal Studies Archives of General Psychiatry 1987 44 540 8 3579500 10.1001/archpsyc.1987.01800180050009

[B67] Hill CE O'Grady KE Elkin I Applying the Collaborative Study Psychotherapy Rating Scale to rate therapist adherence in cognitive-behavior therapy, interpersonal therapy, and clinical management Journal of Consulting and Clinical Psychology 1992 60 73 9 1556289 10.1037//0022-006x.60.1.73

[B68] Hatcher RL Barends AW How a return to theory could help alliance research Psychotherapy: Theory/Research/Practice/Training 2006 43 292 9 10.1037/0033-3204.43.3.292 22122100

[B69] Horvath AO Greenberg LS Development and validation of the Working Alliance Inventory Journal of Counseling Psychology 1989 36 223 33

[B70] Osbourne CA The Role of Alliance and Symptomatic Change Within Cognitive Behaviour Therapy for Depression 2010 Albany: Massey University

[B71] Tichenor V Hill CE A comparison of six measures of working alliance Psychotherapy: Theory, Research, Practice, Training 1989 26 195 9

[B72] Tracey TJ Kokotovic AM Factor structure of the Working Alliance Inventory Psychological Assessment: A Journal of Consulting and Clinical Psychology 1989 1 207 10

[B73] Beck AT Steer R Brown GK Beck Depression Inventory II: Manual 1996 Boston: Hartcourt Brace

[B74] Does van der W BDI-II-NL: Handleiding; De Nederlandse versie van de Beck Depressie Inventory 2002 2 Enschede: Ipskamp

[B75] Whisman M Perez J Ramel W Factor structure of the Beck Depression Inventory-Second Edition (BDI-II) in a student sample Journal of Clinical Psychology 2000 56 545 51 10.1002/(SICI)1097-4679(200004)56:4<545::AID-JCLP7>3.0.CO;2-U 10775046

[B76] Rush AJ Trivedi MH Ibrahim HM Carmody TJ Arnow B Klein DN The 16-item Quick Inventory of Depressive Symptomatology (QIDS), clinician rating (QIDS-C), and self-report (QIDS-SR): A psychometric evaluation in patients with chronic major depression Biological Psychiatry 2003 54 573 83 10.1016/S0006-3223(02)01866-8 12946886

[B77] Rush AJ Carmody T Reimitz PE The Inventory of Depressive Symptomatology (IDS): clinician (IDS-C) and Self-Report (IDS-SR) ratings of depressive symptoms International Journal of Methods in Psychiatric Research 2000 9 45 59 10.1002/mpr.79

[B78] Trivedi MH Rush AJ Ibrahim HM Carmody TJ Biggs MM Suppes T The Inventory of Depressive Symptomatology, Clinician Rating (IDS-C) and Self-Report (IDS-SR), and the Quick Inventory of Depressive Symptomatology, Clinician Rating (QIDS-C) and Self-Report (QIDS-SR) in public sector patients with mood disorders: A psychometric evaluation Psychological Medicine: A Journal of Research in Psychiatry and the Allied Sciences 2004 34 73 82 10.1017/s0033291703001107 14971628

[B79] Derogatis LR Melisaratos N The Brief Symptom Inventory: An introductory report Psychological Medicine: A Journal of Research in Psychiatry and the Allied Sciences 1983 13 595 605 6622612

[B80] Beurs de E Zitman F De Brief Symptom Inventory (BSI). De betrouwbaarheid en validiteit van een handzaam alternatief voor de SCL-90 2005 Leiden: Leids Universitair Medisch Centrum

[B81] Galdón M Durá E Ferrando M Murgui S Pérez S Ibañez E Psychometric properties of the Brief Symptom Inventory-18 in a Spanish breast cancer sample Journal of Psychometric Research 2008 65 533 9 10.1016/j.jpsychores.2008.05.009 19027441

[B82] Graaf de LE Huibers MJH Introductie van de Diagnostic Inventory for Depression in Nederland Tijdschrift voor Psychiatrie 2009 51 675 86 19760567

[B83] Zimmerman M Sheeran T Young D The Diagnostic Inventory for Depression: a self-report scale to diagnose DSM-IV major depressive disorder Clinical Psychology 2004 60 87 110 10.1002/jclp.10207 14692011

[B84] Warshaw MG Dyck I Allsworth J Stout RL Keller MB Maintaining reliability in a long-term psychiatric study: an ongoing inter-rater reliability monitoring program using the longitudinal interval follow-up evaluation Journal of Psychiatric Research 2001 35 297 305 10.1016/S0022-3956(01)00030-9 11591433

[B85] Warshaw MG Keller MB Stout RL Reliability and Validity of the Longitudinal interval follow-up evaluation for assessing outcome of anxiety disorders Journal of Psychiatric Research 1994 28 531 45 10.1016/0022-3956(94)90043-4 7699612

[B86] Graaf de LE Roelofs J Huibers MJH Measuring dysfunctional attitudes in the general population: the DAS-A revised Cognitive therapy and research 2009 33 345 55 10.1007/s10608-009-9229-y 19623267 PMC2712063

[B87] Weissman AN Beck AT Development and validation of the Dysfunctional Attitude Scale; paper presented at the annual meeting of the Association for the Advancement of Behavior Therapy 1978

[B88] Weissmann AN Beck AT Development and validation of the Dysfunctional Attitude Scale; a prelimary investigation 1978 Toronto: O. N.19486732

[B89] Beck AT Brown GK Steer RA Weissmann AN Factor Analysis of the Dysfunctional Attitude Scale in a Clinical Population Journal of Consulting and Clinical Psychology 1991 3 478 83

[B90] Nelson LD Stern SL Cicchetti DV The Dysfunctional attitude scale, how well can it measure depressive thinking? journal of Psychopathology and behavioural assessment 1992 4 217 23

[B91] Oliver M Baumgart EP The dysfunctional attitude scale: psychometric properties and relation to depression in an unselected adult population Cognitive therapy and research 1985 9 161 7 10.1007/BF01204847

[B92] Power MJ Katz R McGuffin P Duggan CF Lam D Beck AT The Dysfunctional Attitude Scale (DAS): A Comparison of Forms A and B and Proposals for a New Subscaled Version Journal of Research in Personality 1994 28 263 76 10.1006/jrpe.1994.1019

[B93] Cohen L Bout van den J Kramer W Vliet van T A Dutch Attributional Style Questionnaire: Psychometric Properties and Findings of Some Dutch-American Differences Cognitive therapy and research 1986 10 665 9 10.1007/BF01173753

[B94] Peterson C Semmel A Baeyer von C Abramson LY Metalsky GI Seligman MEP The attributional style questionnaire Cognitive therapy and research 1982 6 287 300 10.1007/BF01173577

[B95] Molen van der HT Perreijn S Hout van den MA Klinische psychologie: theorieën en psychopathologie 1997 Groningen: Wolters-Noordhoff

[B96] Karpinski A Steinman RB The Single Category Implicit Association Test as a measure of Implicit Social Cognition Journal of Personality and Social Psychology 2006 91 16 32 16834477 10.1037/0022-3514.91.1.16

[B97] Greenwald AG Nosek BA Banaji MR 'Understanding and using the Implicit Association Test: I. An improved scoring algorithm': Correction to Greenwald et al. (2003) Journal of Personality and Social Psychology 2003 85 10.1037/0022-3514.85.2.197 12916565

[B98] Bosson JK Swann WB Jr Pennebaker JW Stalking the perfect measure of implicit self-esteem: The blind men and the elephant revisited? Journal of Personality and Social Psychology 2000 79 631 43 11045743

[B99] Glashouwer KA de Jong PJ Impliciete persoonlijkheidstrekken en psychopathologie: Achtergrond, huidige inzichten en perspectieven Gedragstherapie 2008 41 121 34

[B100] Nosek BA Greenwald AG Banaji MR Understanding and Using the Implicit Association Test: II. Method Variables and Construct Validity Personality and Social Psychology Bulletin 2005 31 166 80 10.1177/0146167204271418 15619590

[B101] Olson MA Fazio RH Relations between implicit measures of prejudice: What are we measuring? Psychological Science 2003 14 636 9 10.1046/j.0956-7976.2003.psci_1477.x 14629698

[B102] Beck AT Weissman A Lester D Trexler L The measurement of pessimism: The Hopelessness Scale Journal of Consulting and Clinical Psychology 1974 42 861 5 4436473 10.1037/h0037562

[B103] Beck AT Steer RA Manual for the Beck Hopelessness Scale 1988 San Antonio, TX: Psychological Corporation

[B104] Dyce JA Factor structure of the Beck Hopelessness Scale Journal of Clinical Psychology 1996 52 555 8 10.1002/(SICI)1097-4679(199609)52:5<555::AID-JCLP10>3.0.CO;2-D 8877692

[B105] Young MA Halper IS Clark DC Scheftner WA An item-response theory evaluation of the Beck Hopelessness Scale Cognitive Therapy and Research 1992 16 579 87 10.1007/BF01175143

[B106] Horowitz LM Rosenberg SE Baer BA Ureño G Villaseñor VS Inventory of Interpersonal problems: Psychometric properties and clinical applications Journal of Consulting and Clinical Psychology 1988 56 885 92 3204198 10.1037//0022-006x.56.6.885

[B107] Beurs de DP Further validation of the Leiden Index of Depression Sensitivity Tijdschrift voor Psychiatrie; samenvattingen 37ste voorjaarscongres 2009 supplement 121674452

[B108] Does van der W Cognitive reactivity to sad mood: structure and validity of a new measure Behaviour Research and Therapy 2002 40 105 20 10.1016/S0005-7967(00)00111-X 11762423

[B109] Nolen-Hoeksema S Morrow J A prospective study of depression and posttraumatic stress symptoms after a natural disaster: The 1989 Loma Prieta earthquake Journal of Personality and Social Psychology 1991 61 115 21 1890582 10.1037//0022-3514.61.1.115

[B110] Raes F Hermans D Eelen P Kort instrumenteel De Nederlandstalige versie van de Ruminative Response Scale (RRS-NL) en de Rumination on Sadness Scale (RSS-NL) Gedragstherapie 2003 36 97 104

[B111] Tafarodi RW Swann WB Two-dimensional self-esteem: Theory and measurement Personality and Individual Differences 2001 31 653 73 10.1016/S0191-8869(00)00169-0

[B112] Vandromme H Hermans D Spruyt A Eelen P Dutch translation of the Self-Liking/Self-Competence Scale - Revised: A confirmatory factor analysis of the two-factor structure Personality and Individual Differences 2007 42 157 67 10.1016/j.paid.2006.07.001

[B113] Drummond MF Schulpher MJ Torrance GW O'Brien B Stoddart GL Methods for the Economic Evaluation of Health Care Programmes 2005 Oxford: Oxfor University Press

[B114] Graaf de LE Gerhards S Evers S Arntz A Riper H Severens J Clinical and cost-effectiveness of computerised cognitive behavioural therapy for depression in primary care: Design of a randomised trial BMC Public Health 2008 8 224 10.1186/1471-2458-8-224 18590518 PMC2474681

[B115] Goossens MEJB Rutten-van MPMH Mölken Vlaeyen JWS van der Linden SMJP The cost diary: a method to measure direct and indirect costs in cost-effectiveness research Journal of Clinical Epidemiology 2000 53 688 95 10.1016/S0895-4356(99)00177-8 10941945

[B116] Hakkaart-van Roijen L Trimbos/iMTA questionnaire for Costs associated with Psychiatric Illness (TiC-P) 2002 Rotterdam: Institute for medical Technology Assessment, Erasmus University

[B117] van Asselt ADI Dirksen CD Arntz A Giesen-Bloo JH van Dyck R Spinhoven P Out-patient psychotherapy for borderline personality disorder: Cost-effectiveness of schema-focused therapy v. transference-focused psychotherapy British Journal of Psychiatry 2008 192 450 7 10.1192/bjp.bp.106.033597 18515897

[B118] Koopmanschap M Meerding W Evers S Severens J Burdorf A Brouwer W Handleiding voor het gebruik van PRODISQ. Een modulaire vragenlijst over de relatie tussen ziekte en productiviteitskosten. Toepasbaar bij economische evaluaties van gezondheidszorgprogramma's voor patiënten en werknemers 2004 Rotterdam/Maastricht: Erasmus Universiteit Rotterdam/Erasmus Medisch Centrum/Universiteit Maastricht

[B119] Mundt JC Marks IM Greist JH Shear K The Work and Social Adjustment Scale: A simple accurate measure of impairment in functioning British Journal of Psychiatry 2002 180 461 4 10.1192/bjp.180.5.461 11983645

[B120] Mataix-Cols D Cowley AJ Hankins M Schneider A Bachofen M Kenwright M Reliability and validity of the Work and Social Adjustment Scale in phobic disorders Comprehensive Psychiatry 2005 46 223 8 10.1016/j.comppsych.2004.08.007 16021593

[B121] EuroQolGroup EuroQol -- a new facility for the measurement of health-related quality of life Health Policy 1990 16 199 208 10109801 10.1016/0168-8510(90)90421-9

[B122] van der Zee KI Sanderman R Het meten van de algemene gezondheidstoestand met de RAND-36: een handleiding 1993 Groningen: Noordelijk Centrum voor Gezondheidsvraagstukken

[B123] Martin DJ Garske JP Davis MK Relation of the therapeutic alliance with outcome and other variables: a meta-analytic review Journal of Consulting and Clinical Psychology 2000 68 438 50 10883561

[B124] Horvath AO Bedi RP Norcross JC The alliance Psychotherapy relationships that work: Therapist contributions and responsiveness to patients 2002 New York: Oxford University Press 37 69

[B125] Vervaeke GAC Vertommen H De Werkalliantievragenlijst (wav) Gedragstherapie 1996 2 139 44

[B126] Goldberg TE Weinberger DR Genes and the parsing of cognitive processes Trends in Cognitive Sciences 2004 8 325 35 10.1016/j.tics.2004.05.011 15242692

[B127] Domschke K Braun M Ohrmann P Suslow T Kugel H Bauer J Association of the functional -1019C/G 5-HT[sub]1A[/sub] polymorphism with prefrontal cortex and amygdala activation measured with 3 T fMRI in panic disorder International Journal of Neuropsychopharmacology 2006 9 349 55 10.1017/S1461145705005869 16316476

[B128] Pezawas L Meyer-Lindenberg A Drabant EM Verchinski BA Munoz KE Kolachana BS 5-HTTLPR polymorphism impacts human cingulate-amygdala interactions: A genetic susceptibility mechanism for depression Nature Neuroscience 2005 8 828 34 10.1038/nn1463 15880108

[B129] Smolka MN Schumann G Wrase J Grusser SM Flor H Mann K Catechol-O-Methyltransferase val158met Genotype Affects Processing of Emotional Stimuli in the Amygdala and Prefrontal Cortex Journal of Neuroscience 2005 25 145 59 10.1523/JNEUROSCI.1792-04.2005 PMC6725630 15673663

[B130] Jacobson NS Truax P Kazdin AE Clinical significance: A statistical approach to defining meaningful change in psychotherapy research Methodological issues & strategies in clinical research 1992 Washington, DC US: American Psychological Association 631 4821676575

[B131] Evans C Margison R Barkham M The contribution of reliable and clinically significant change methods to evidence-based mental health Evidence-Based Mental health 1998 1 70 2 10.1136/ebmh.1.3.70

[B132] Nock MK Conceptual and design essentials for evaluating mechanisms of change Alcoholism: Clinical and Experimental Research 2007 31 4S 12S 10.1111/j.1530-0277.2007.00488.x 17880341

[B133] Haaga DAF Stiles WB Snyder CR, Ingram RE Randomized clinical trials in psychotherapy research:Methodology, design, and evaluation Handbook of psychological change: Psychotherapy processes and practices for the 21st Century 2000 New York: Wiley 14 39

